# Embalmment

**Published:** 1857-08

**Authors:** W. T. Cox

**Affiliations:** West Feliciana, La.


### *Embalmment. Burial.*

West Feliciana, La., March 28th, 1857.

Dr. Dowler :—*Dear Sir :*—The circumstances of the body of Dr. Kane having been "embalmed after the method of Gannal," has raised the question in my mind as to what that method is ? I am aware that it purports to be, the injection into the arteries of the acetate of alumine, formed by mixing solutions of the sulphate of alumina and potassa, and the acetate of lead, and throwing down the insoluble sulphate of lead, and forming the acetate of alumine, yet he admits that this preparation, in the course of time, causes the subject to take on "all the exterior aspect of the negro," owing to the lead still held in solution combining with the hydro-sulphuric acid disengaged by the decomposition of the body; in fact, this process seems only intended for the preservation of bodies for dissection, as he acknowledges in more than one place, that he retains the secret of embalming for his own special use and benefit. As my copy of Gannal's work on the subject dates back to the year 1840, and only gives (or pretends to give) his researches up to 1838, and that in a very (to me) obscure manner, my information may be old ; and for this reason, I am induced to ask whether or not his researches have been continued, according to promise, and if so, what is the result ? In fine, what is the method of Gannal ? and does any one really possess a knowledge of a method of preserving the bodies of the dead for any length of time in any climate, except those known to possess the property independently of any artificial process whatever ? and if so, what is the method ? An answer to these queries through the columns of the New Orleans Medical and Surgical Journal, would be particularly gratifying to me, and, no doubt, interesting to many of your readers. Respectfully yours, etc.,

W. T. COX, M. D.

——" Embalm me,

Then lay me forth."—Shaks. Henry VIII.

——" Thy virtues are
The spices that embalm thee ; thou art far
More richly laid, and shalt more long remain
Still mummified within the hearts of men
Than if to lift thee in the rolls of fame
Each marble spoke thy shape, all brass thy name."—Hall.

The *Gazete Médicale de Paris*, No. 46, *anno* 1851, contains a report on the substances used in embalming the dead. This report is an official one made by a commission appointed by the Academy of Medicine, and is an answer to the request made by the Minister of Agriculture and Commerce; the latter having consulted the academy agreeably to the advice of the council of health, in regard to the interdiction of embalming with poisonous substances. The commission consisting of MM. Orfila, Bussy, Chevallier, Poiseille, and the reporter, M. Caventou. The result of their deliberations may be summed up in the following dense translation: The recognized agents, adapted to the preservation of animal bodies, as proven by numerous experiments, are partly derived from the mineral kingdom; such are certain alkaline and earthy salts, as the chlorides of potassium or sodium, the sulphate of soda, the nitrate of potass, the aluminous salts, etc., but all such are quite limited in their preservative powers, unless associated with arsenic. The use of the metalic salts, such as those of zinc, mercury, lead, copper or iron, is, to a certain extent, poisonous. Arsenical preparations have been already proscribed by a law passed October 29, 1846, since which, corrosive sublimate has been much used; also the acetate of lead and the salts of copper. The dangers incidental to the employment of these poisons, ought, as the commission think, to exclude them from use in embalming the dead.

M. Bussy thought the report too absolute in maintaining that no substance devoid of arsenic can be relied on. M. Gannal affirmed the contrary, and M. Chevallier, who had examined a great number of preparations made and intended for embalming the dead, found that they contained no arsenic. M. Bussy thought that the salts of alumina which are convenient and useful for embalment should not be interdicted.

M. Caventou denied the efficacy of aluminous salts in embalming, unless the body be buried in the earth. M. Bussy, however, said that he would not oppose the adoption of the report.

M. Orfila said he had examined M. Gannal's preservative preparations, which he found not *lightly* but *very strongly charged* with arsenic. After having examined M. Gannal's first preparation for embalming, M. G. directed the commission to a second kind which contained no arsenic, but which did not preserve the body from running into horrible rottenness: "*Les cadavres injectés avec ce liquide furent trouvés, au bout de quelque temps, horriblement pourris.*" M. Orfila denied the efficacy of aluminous salts for the purpose of embalming the dead.

The Academy adopted the conclusions of the report, (Nov. 11, 1851.)

Some time subsequently to this report of the Academy, the following statement concerning M. Gannal's reputed discovery appeared in the public journals:

"*Verification of an Interesting Discovory.*—Dr. Quesneville, the editor of the *Revue Scientifique* of Paris, in a late number of that journal gives, an account of some experiments, at which he was present, to test the merits of a new discovery in the art of embalming. 'Agreeably to the invitation,' he says, 'which was addressed to us in common with a large number of physicians and journalists, we were present at the exhumation of a body that had been embalmed after the method of Gannal. This took place in the cemetery of Père La Chaise, and we were attracted by no ordinary feeling of mere curiosity. A recent report of the Academy of Medicine had called in question the reality of this discovery, supposed indisputably established. A public defiance had been thus given to this celebrated embalmer, who was not slow in taking up the glove thus thrown to him. We found on the spot, a large concourse of curious spectators; but the family would not consent to admit more than six physicians out of more than 150, who came to ascertain the result of this curious exhumation. The body in question, embalmed in 1844, was found in a perfect state of preservation. Thus have failed, therefore, these inexplicable efforts of this learned body, after so short a time, to destroy the reputation of a discovery extolled by their own published testimony.' "

Embalming, which is very rarely practised in the United States, is deemed of great concernment in some European countries.

" *Embalming in France.*—It is a singular fact," says the London *Lancet*, " but nevertheless true, that in France, at the present time, (1844) the higher and richer classes are nearly as much in the habit of embalming their departed relations as were the Egyptians, or the inhabitants of the Canary Islands, in the days of old. The principal difference, however, between the men of former times and our continental neighbors is, that the former embalmed their dead in such a manner as to render them nearly imperishable, as our museums can testify, under the impression that the soul remained near the body as long as it retained its terestrial form; whilst the latter, who are not much troubled with superstition, as every one knows, are content to impart to their dead a temporary immunity from decay."

G. R. Gliddon, Esq., formerly U. S Consul at Cairo, an able Egyptologist, long a resident in Egypt, having left me for perusal, a MS. of his on Mummies, I have extracted from it the following abridged statements of facts and opinions: " The soil of Egypt is so profoundly drenched by inundation, that from the earliest to the latest times, no cemeteries were constructed on the alluvial plain. The dead were interred upon the *sand* at a level high above the river's utmost rise, where they rapidly *dried.* The towns were situated some miles off from the *Necropoles.* The dead were carried to the desert, which in some cases lay ten to fifteen miles off, and which was used as the primitive burial places before the art of mummification was introduced—before pyramids and catacombs were constructed.

" In summer the plague does not occur along the Nile—it is a winter and spring disease that dies invariably in *June.* But the peculiar atmospheric phenomena of Egypt are then, such, that as the heat is greatest so the N. W. or Etesian blows highest. In fact, what with the N. W. wind blowing up the river eight months of the year, and the S. E. blowing four months down, the valley of the Nile is a perfect *air funnel* through which for six months out of the twelve the wind blows a gale upwards or downwards.

" We return to the earliest days of Nilotic ante-monumental history when the primitive wandering shepherd transformed by time and necessity into a stationary farmer, had witnessed the drying effects of the sands of the desert, impregnated with *Natron*, which preserved the dead from putrefaction; he was led to use this and *baking* or *desiccation*, bitumen, bandages, clothes, etc., and to excavate rocks and make tombs for his dead.

" Mummification ceased about A. D. 640. The Necropoles of Memphis covers about twenty-two miles from N. to S. and about a mile in breadth from E. W., being the most ancient extant.

"The largest Necropoles of the Nile are to be found on the *Western* side along the Lybian chain of limestone hills, in the neighborhood of the larger cities. Sauàra, the nearest to Memphis, is the largest. From the remote age of *Menes*, (whose reign cannot be brought down later than 3600 B. C., probably 5000 B. C.,) the city of Memphis continued to pour out her dead upon this vast emporium of bodies, down to from 500 to 800 A. D.

"Great inconvenience is experienced, now-a-days, in Egypt, owing to the heat and rapid putrefaction of *corpses* which are in consequence hurried to the grave with a celerity quite appalling to our European ideas. In the plague of 1835, I spoke with a Nubian servant who was apparently in good health about 12, M., and by 2 o'clock his bier passed my house with the lugubrious chant announcing his body's last journey to the dust. Cases such as these are familiar to all in oriental countries. At Cairo I have frequently had occasion to pay the last obsequies to friends whose relations wished the *corpse* to be sent to the Protestant burial ground at Alexandria, to England, and even to America, and with the utmost expedition, and at lavish expense, in copper or leaden coffins, it was scarcely possible to place the corpse into them quickly enough to escape inconveniences: for without *ice* it is impossible.

"The invention of mummification obviated all difficulties: the bodies went instantly to the embalmers as soon as the breath had abandoned them; every provincial temple had its complete establishment for converting them into mummies at any expense the family chose, and after about seventy days the corpses were ready to be conveyed any distance to the tombs."

Although the period of seventy days here given agrees with classical history, (Herodotus and others) yet the Pentateuchal history proves that Jacob's body which was embalmed in Egypt, agreeably to the method then in use in that country, required but forty days to "fulfil" the whole term "of those who are embalmed:" "And Joseph commanded his servants, the physicians, to embalm his father: and the physicians embalmed Israel. And *forty days* were fulfilled for him; for so are fulfilled the days of those which are embalmed."—*Gen. L.*, 2, 3.

Embalmment among the Hebrews at a later era is thus briefly indicated in sacred history: "And there came also Nicodemus (which at the first came to Jesus by night) and brought a mixture of myrrh and aloes, about an hundred pound weight. Then took they the body of Jesus, and wound it in linen clothes with the spices, as the manner of the Jews is to bury."—*St. John* xiq, 39, 40.

Mr. Gliddon estimates that the Necropoles of Memphis and Thebes held half of the dead of ancient Egypt below the first cataract, and that in 3,000 years, during which mummification prevailed, the number of mummies amounted to four hundred and fifty or five hundred millions, "all being imperishable save by the hand of *man*."

The average length of mummies, owing partly to the desiccation each body has undergone, which has contracted its true proportions, and partly to the fact that the ancient Egyptians were not a large race of men, may be roughly estimated at five and a half feet, when enveloped in their wrappers. The height of the reclining body and the breadth across the shoulders one and a half feet each.

"Extraordinary as my assertion may seem, the tombs of Egypt excavated in the rock, are numerous enough to hold all these mummies.

"In chronological duration, *mummification* antedates all human history, all monumental records, and following its phases down to the fifth century after our era, a period exceeding five thousand years!"

In a published work, (*Otia Ægyptiäca*) Mr. Gliddon gives the prices of Egyptian Embalments: "Three classes of mummies; the first of which costs one thousand two hundred and fifty dollars, the second, three hundred dollars, and the third, or cheapest, twenty dollars; some having one thousand yards of *linen* weighing forty-six pounds, varying in texture from good calico to superfine cambric.

"The Egyptians were under our average size. The length of life in Egypt, even in days long before Abraham, being the same as our own, proved by innumerable sepulchral tablets, the reigns of kings, and the skulls of mummies."

The Pharaonic embalmers and their successors and imitators, down to M. Gannal, stand at an almost infinite distance below the late celebrated Italian, Segato, who possessed the art of fossilizing or petrifying the human body, not excepting its softest tissues, as the viscera, brain, etc.—There is, as reliable travelers assert, a table in the museum of Florence, made in mosaic out of the human organs, solid like marble, the pieces of which were prepared by Segato. He carried his secret with him to the tomb.

The reader is referred to an article in this Journal, translated by Dr. M. M. Dowler from the *Jour. de Méd. de Bourdeaux*, (July, 1856) in which will be found Prof. Barbet's account of the method by which M. Lapeyrouse mineralizes animal matters by the earthy chlorides in twenty to thirty hours, so that animal substances will remain unchanged for "an unlimited time." This process, M. Lapeyrouse has patented. His experiments were made before the commission of the Council of Hygiene of Gironde, and should future experience and the test of time confirm all that Prof. Barbet of Bordeaux has said in its favor, the discovery will not only supersede all other methods of embalming the dead and of preparing anatomical subjects for dissection or preservation, bnt must prove highly advantageous in an industrial and economical point of view, and the more so, because it is not attended with much expense.

The value of this discovery, if discovery it be, cannot be definitely proven, except by the test of time. This test is in favor of the Egyptians as yet, seeing that without having used poisons dangerous to the living, they have preserved the dead for thousands of years. MM. Gannal, Lapeyrouse and others, must perfect their methods by excluding deadly poisons, and by awaiting the lapse of the forty centuries which Napolean saw perched upon the pyramids, looking down upon the army at the battle of the pyramids!

Baron Larrey, the great surgeon, in his Military Memoirs of the campaigns in Egypt, gives a very interesting account of the Egyptian mode of embalming, yet he claims, nevertheles, for the moderns, the highest degree of success in this behalf, giving at the same time his mode, which consists chiefly in the application of corrosive sublimate, to the fleshy parts, and then the plunging of the whole body in a solution of this dangerous poison for ninety or a hundred days. "Thus," says he, "I preserved the body of Col. Morlan, who fell at the battle of Austerlitz; it is still perfect." But it is poisoned! To prepare, to dissect, or to keep such a subject is not devoid of danger. Should any evil minded person wish to poison his neighbor, a bit of the gallant colonel might be used. Besides, religion or affection, which gives rise to the embalment of the dead, must abhor the association of the idea of a beloved friend with a deadly poison!

The following paper from the *Charleston Medical Journal* translated from the *Moniteur des Hôpitaux*, presents a brief historical summary of the art of embalming:

We must go back to the earliest ages, in order to find the origin of preserving bodies, but for its history we must confine ourselves to those traditions which have been handed down to us in connection with the discovery of monuments which have escaped the destructive effects of time. Among the nations of Asia and Africa, where embalming appears to have been a general custom, those holding the first position were the Egyptians and the inhabitants of India. The Egyptians particularly, who left such numerous traces of ancient splendor, seemed to have wished to perpetuate themselves even in death, in strewing upon their soil mummies as indestructible as the superb monuments which concealed them.

Historians and antiquarians still conjecture on the motive which led these people to preserve the dead with so much care. Some attribute it to the belief that the soul, after escaping from the body, wandered about during 3,000 years to reënter it, and that, therefore, the destruction of the former would compel it to pass into the body of an animal. The more rational believe the practice to have arisen in connection with the principles of hygiene, one of the branches of medicine that the Egyptians cultivated with so much success. For in these hot regions, only receiving fertility from the overflowing of the Nile, the decomposition of bodies deposited in the earth would soon destroy the purity of the air, and spread among the population the seed of the most virulent disease. It is true that the places destined for burial were above the inundations of the river, but in these elevated places the putrefaction of bodies would have been even more fatal; for the winds which prevail in these countries in bringing putrid miasms from a distance would have transported also their disastrous effects. These considerations were too intimately connected with the interests of the public health to escape the enlightened spirit of those who had it under care; thus, Herodotus relates, that during a period of three thousand years Egypt was one of the healthiest countries in the world. Now, subject to the fatal yoke of Mahometanism, it no longer enjoys this immunity, but it has become the hot-bed of the plague. The various modes of embalming in Egypt might be reduced to the following operations:

1st. Remove from the body all fatty matters and mucous portions, by the prolonged action of soda.

2d. After having well washed the body, dry it in the air or in a stove.

3d. Preserve it by employing bitumen, balsams, resins and salts.

4th. Surrounding it with numerous strips of cloth, smeared with gum or bitumen.

The aromatics employed by the rich, were myrrh, aloes, cannella and cassia. For the inferior classes, cedar and the bitumen of Judea.

The duration of embalming varied from forty to seventy days, depending much on the drying of the bodies. When the operation was finished, they were enclosed in sarcophagi, and deposited in sepulchral chambers, inaccessible to moisture, the temperature being maintained at about 88 degrees, Fahr.

It is under these favorable conditions that a great number of mummies have been preserved through a long series of ages, and now supply us with sufficiently accurate knowledge of the art of embalming among the ancient Egyptians.

The Indian mummies, exhibited at the Garden of Plants, appear to have undergone an analogous preparation to those of Egypt. After embalming, the bodies were sewed up in the skin of goats, and deposited in catacombs.

In examining carefully the tissues of mummies, an analysis will detect nitrate or carbonate of potash, or sometimes sulphate and chloride of soda, or the iodides of lime and magnesia. During the infancy of the art, drying and aromatic substances were alone employed; later, saline matters entered among the ingredients. Ethiopians, inhabiting a country richer in gum than the rest of the globe, were accustomed to enclose their body in a molten mass of this transparent matter, while the Scythians and Persians covered them with an envelope of wax.

Pliny speaks of the antiseptic properties of honey, and it is related that Alexander the Great, after death, was rubbed with honey before burial.

Modern nations following the example of the ancient Egyptians have long practised evisceration in connection with the use of a number of solid and fluid substances to preserve bodies. Alcohol, essential oils, and compound liniments are most conspicuous; balsamic and aromatic powders with saline substances are also used.

In the middle ages the art of embalming consisted in mixing aromatic substances with salt, with which the bodies were filled. Henry I. of England was thus embalmed in 1135: long incisions were made in various parts of the body, filled with this composition, then sewed up, the body being then enveloped in a beef's skin and enclosed in a coffin. The employment of salt for the preservation of the bodies of kings, is well known in history, the sellers of salt claiming as their right to assist at the royal funerals, and bear the bodies of the kings.

In 1658, Louis C. Bils, a noble of Holland, well skilled in anatomy, announced that he had found a way of preserving bodies from putrefaction without evisceration, so that the form and flexibility of the extremities being retained they could be used for dissection. The announcement of this discovery on the part of the first noble who had given up himself to the pursuit of anatomy made a great sensation. At the height of his renown the States of Brabant bought from him five embalmed subjects for 22,000 florins. Zipœus, professor of anatomy at the University of Louvain, to whom they were given, was appointed to receive the secret; but a few weeks had hardly elapsed when the bodies became putrid. Bils, pretending that such a result was owing to the jealousy of the professors who placed his preparations in a damp situation, in order to promote decomposition. Bils' treatment of bodies was with himself eminently successful; the secret was buried with him.

Ruysch, also a Dutch physician and anatomist of celebrity, tried to eclipse his adversary, Bils. He succeeded in injecting pieces, which preserved their softness, flexibility and color. His collection so attracted general attention that it was visited by all the curious of Europe. It is said that Peter the Great during a visit to this museum, was so attracted by the embalmed body of a little child, which appeared to invite him with a smile, that he kissed it. Ruysch sold his collection, at the entreaties of Peter the Great, for 30,000 florins. Although seventy-nine years old, he immediately recommenced forming a collection, which he succeeded in doing in two years. In dying, in 1731, he also carried off with him the secret of his admirable injections.

Darconville was the first who discovered, in 1762, the preservative properties of corrosive sublimate, but we are indebted to the illustrious Chaussier for the practical use of this drug in preserving animal matter.

Beclard, chief of the anatomical works of the faculty of medicine of Paris, applied the sublimate in embalming bodies. Charged with preserving the body of a young man who died with hectic fever, (the parents refusing to have the body opened,) after making numerous punctures and incisions in every portion of the body, he placed it in a solution of corrosive sublimate, in which it was kept for two months. When taken out, it was dried for a few days, and then enclosed in a glass case, where it remained for a year without smell, or the slightest appearance of alteration. It was then given to the family. The skin was discolored grayish, and the features were somewhat changed from the thinning of the lips, cheeks, eyelids and ears.

Bugliaretti, an Italian physician, united arsenic with sublimate. He injected with this solution the primitive carotid artery, the right jugular vein, the external iliac on both sides, and by using a trocar, he forced the fluid into the thorax and abdomen. The results obtained, appeared to be very similar to the preceding.

Dr. Tranchina, of Naples, acquired a great reputation in Italy for preserving bodies. His method consisted in an injection of a solution composed of 4 lbs. of arsenic in 10 lbs. of water. This mode of preservation, very dangerous for dissectors, did not serve the purpose of embalming, for the body became livid and atrophied in drying, till only a skeleton remained, covered with skin from which the cuticle had peeled.

In 1822, M. Gannal, manufacturer of glue, discovered that a solution of salt and alum would prevent fermentation. With this mixture in connection with a small quantity of arsenite of soda, he injected the body of a child, which was left on the tables of the Morgue for three months, and from which he attained a great reputation.

It is nearly fifty years since chloride of zinc was first used in England for preserving animal matters. Sucquet took out a patent for preserving pieces, by first injecting them with sulphate of soda, and then plunging them in a solution of chloride of zinc.

M. Granger had been previously acquainted with the antiseptic properties of the sulphate of zinc, and a young savant, M. Gratiolet, conservator of comparative anatomy at the Garden of Plants, had tried it for preserving anatomical pieces. After numerous experiments he abandoned this salt, which did not preserve sufficiently, as the tissues became discolored. The skin resembled parchment, and the muscles diminished more than a third of their volume. Although injections of salt tried at the anatomical rooms in Paris were unsuccessful, it is still used by anatomists in preserving subjects.

Dr. Roux, of Nimes, teaches the following system : It is impossible to find an antiseptic, which will preserve all subjects. The following circumstances should be taken into consideration : 1st. The constitution of the subject. 2d. The cause of death. 3d. The temperature. Anatomists must have daily observed in the dissecting room, that putrefaction is differently produced : in some subjects it shows itself with extreme rapidity, in others, some days elapse, and a few might be kept for even weeks, without much decomposition. From this fact, he concludes that the choice of an antiseptic agent depends upon the character of the substance which it is intended to preserve—that is to say, upon each subject—should be chemically treated according to the constitution, cause of death, and influence of temperature. After long experience, this anatomist lays down the following rules :

A young animal is best preserved by using a sulphate ; a sulphite for an animal at puberty ; and a chloride for an adult ; and lastly, to prevent mould from appearing on the surfaces pour over them either some essential oil, ether, or chloroform. There is no universal antiseptic agent. By following these rules, astonishing results will be obtained.

*Preservation of Human Bodies.*—No people have succeeded so well as the ancient Egyptians in preserving the bodies of their friends and relatives from decomposition. We have been at the vast Necropolis of Sakkara, and watched the process of dragging up the mummies from the deep pits in which they had rested for at least three thousand if not four thousand years. The restless Bedouin Arabs have been carrying on the revolting occupation of rifling those deep, capacious vaults in which many hundred bodies were artistically packed, several centuries, for the sake of the jewelry, shoes, caps and specimens of ancient arts that are thus brought to light ; and although the business is still actively pursued, it is hardly probable that there will be any apparent diminution of mummies a thousand years hence.

This not merely demonstrates the denseness of the Nilotic population through a long series of Pharaonic ages, but also the universal custom of embalming all the dead. Indeed, the same care bestowed in the mummification of human beings in Egypt, whether from religious or hygienic views, was also extended to dogs, cats, birds, crocodiles, etc., so that countless millions will not over express the number still remaining in the most perfect state of preservation, a proof of all that might be collected on this curious and interesting subject.

A strong desire was evinced both in France and England, to prevent the decay of their early sovereigns, but their efforts, based upon no scientific principles, were very imperfect, so that it was quite rare to find a well-preserved body in any of the royal vaults in Europe. Generally they are in deep, damp places, under floors, or beneath low, massive arches, where no rays of sunlight ever appear, to dissipate the sweating moisture that corrupts whatever is placed within the gloomy resting-places of kings and potentates.

The royal vaults in which the whole line of electors, emperors, empresses and the families of the rulers of the Austrian empire are placed for the sleep of death, are dry, tolerably light and admirably ventilated.—What the condition of the bodies may be in their costly sarcophagic and metallic coffins is of course unknown, as no mention is extant of any explorations for ascertaining.

The sarcophagus in which the august remains of Maria Theresa were laid—which was constructed under her own eyes, at an enormous cost, is a massive metallic structure occupying as much space on the floor as a large-sized bedstead. It is about five feet high, having magnificent metallic statuary at the corners, with curiously draped and winged figures as accompaniments. The Duke of Reichstadt, the son of Napoleon le Grand, is in a plain, unadorned metallic coffin, resting on a stool at the side of the wall. It has a dull metallic appearance, like tarnished iron. Possibly it may be zinc.

The Empress Louisa Maria, the second wife of Napoleon, the mother of Reichstadt, who died reigning Duchess of Parma, is in a similar looking coffin, placed in a like position a few feet from her son.

But of all the modern final reposing places of royalty we have personally examined, those of the Sultans of Turkey are the most gorgeous and extraordinary in all respects. It would require a more severe examination of packed-away manuscripts, written over the mouldering remains of those ferocious monsters,—from Mahomet the Second to Mahomoud the Second, the father of the present Pedisha, Abdel Mejid, than we care to undertake, for particulars, and thus we close these hasty observations by showing the condition of some of the kings of England during eventful historical periods.

The English evidently desired to so protect the bodies of their early and later kings that they should resist the chemical tendency to decomposition. They partially succeeded in a few instances, only, as will be noticed in the following collection of facts.

King Edward I. died in July, 1307, and notwithstanding his injunctions, was buried in Westminster Abbey in October of the same year. It is recorded that he was embalmed, and orders for renewing the cere-cloth about his body were issued in the reigns of Edward III. and Henry IV. The tomb of this Monarch was opened, and his body examined in January, 1774, under the direction of Sir Joseph Ayloffe, after it had been buried four hundred and sixty-seven years. The following account is extracted from a contemporaneous volume of the Gentleman's Magazine:

"Some gentlemen of the Society of Antiquarians, being desirous to see how far the actual state of Edward First's body answered to the methods taken to preserve it, obtained leave to open the large stone sarcophagus, in which it is known to have been deposited, on the north side of Edward the Confessor's chapel. This was accordingly done on the morning of January 2, 1774, when in a coffin of yellow stone they found the royal body in perfect preservation, inclosed in two wrappers; one of them was of gold tissue, strongly waxed, and fresh, the other and outermost considerably decayed. The corpse was habited in a rich mantle of purple, paned with white, and adorned with ornaments of gilt metal, studed with red and blue stones and pearls. Two similar ornaments lay on the hands. The mantle was fastened on the right shoulder by a magnificent *fibula* of the same metal, with the same stones and pearls. His face had over it a silken covering, so fine, and so closely fitted to it, as to preserve the features entire. Round his temples was a gilt cornet of fleurs de lys. In his hands, which were also entire, were two sceptres of gilt metal; that in the right surmounted by a cross fleure, that in the left by three clusters of oak leaves, and a dove on a globe; this sceptre was about five feet long. The feet were enveloped in the mantle and other coverings, but sound, and the toes distinct. The whole length of the corpse was five feet two inches."

Edward I. died at Burgh-upon-Sands, in Cumberland, on his way to Scotland, July 7, 1307, in the sixty-eighth year of his age.

Another instance of partial preservation, is that of the body of King Charles I., who was beheaded by his subjects in 1649. The remains of this unfortunate monarch are known to have been carried to Windsor, and there interred by his friends without pomp, in a hasty and private manner. It is stated in Clarendon's History of the Rebellion, that when his son, Charles II., was desirous to remove and re-inter his corpse at Westminster Abbey, it could not by any search be found. In constructing a mausoleum at Windsor in 1813, under the direction of George IV., then Prince Regent, an accident led to the discovery of this royal body. The workmen, in forming a subterraneous passage under the choir of St. George's Chapel, accidentally made an aperture in the wall of the vault of King Henry VIII. On looking through this opening it was found to contain three coffins, instead of two, as had been supposed. Two of these were ascertained to be the coffins of Henry VIII. and of his queen, Jane Seymour. The other was formally examined, after permission obtained, by Sir Henry Halford, in presence of several members of the royal family, and other persons of distinction. The account since published by Sir Henry, corroborates the one which had been given by Mr. Herbert, a groom of King Charles's bedchamber, and is published in Wood's Athenæ Oxoniensis.

"On removing the pall," (says the account,) "a plain leaden coffin presented itself to view, with no appearance of ever having been inclosed in wood, and bearing an inscription, 'King Charles, 1648,' in large, legible characters, on a scroll of lead encircling it. A square opening was then made in the upper part of the lid, of such dimensions as to admit a clear insight into its contents. These were, an internal wooden coffin, very much decayed, and the body carefully wrapped up in cere-cloth, into the folds of which a quantity of unctuous matter, mixed with resin, as it seemed, had been melted, so as to exclude, as effectually as possible, the external air. The coffin was completely full, and, from the tenacity of the cere-cloth, great difficulty was experienced in detaching it successfully from the parts which it enveloped. Wherever the unctuous matter had insinuated itself, the separation of the cere-cloth was easy; and where it came off, a correct impression of the features to which it had been applied, was observed. At length the whole face was disengaged from its covering. The complexion of the skin of it was dark and discolored. The forehead and temples had lost little or nothing of their muscular substance; the cartilage of the nose was gone; but the left eye, in the first moment of exposure, was open and full, though it vanished almost immediately; and the pointed beard, so characteristic of the period of the reign of King Charles, was perfect. The shape of the face was a long oval; many of the teeth remained; and the left ear, in consequence of the interposition of the unctuous matter between it and the cere-cloth, was found entire.

"It was difficult, at this moment, to withhold a declaration that, notwithstanding its disfigurement, the countenance did bear a strong resemblance to the coins, the busts, and especially to the picture of King Charles the First, by Vandyke, by which it had been made familiar to us. It is true that the minds of the spectators of this interesting sight were well prepared to receive this impression; but it is also certain that such a facility of belief had been occasioned by the simplicity and truth of Herbert's narrative, every part of which had been confirmed by the investigation, so far as it had advanced; and it will not be denied that the shape of the face, the forehead, the eyes, and the beard, are the most important features by which resemblance is determined.

"When the head had been entirely disengaged from the attachments which confined it, it was found to be loose, and without any difficulty was taken out and held up to view. The back part of the scalp was entirely perfect, and had a remarkably fresh appearance; the pores of the skin being most distinct, and the tendons and ligaments of the neck were of considerable substance and firmness. The hair was thick at the back part of the head, and in appearance nearly black. A portion of it, which has since been cleaned and dried, is of a beautiful dark brown color. That of the beard was a redder brown. On the back part of the head it was not more than an inch in length, and had probably been cut so short for the convenience of the executioner, or perhaps by the piety of friends soon after death, in order to furnish memorials of the unhappy king.

"On holding up the head, to examine the place of a separation from the body, the muscles of the neck had evidently retracted themselves considerably; and the fourth cervical vertebra was found to be cut through its substance transversely, leaving the surfaces of the divided portions perfectly smooth and even, an appearance which could have been produced only by a heavy blow, inflicted with a very sharp instrument, and which furnished the last proof wanting to identify King Charles the First."

The foregoing are two of the most successful instances of posthumous preservation. The care taken in regard to some other distinguished personages has been less fortunate in its result. The coffin of Henry VIII. was inspected at the same time with that of Charles, and was found to contain nothing but the mere skeleton of that king. Some portions of beard remained on the chin, but there was nothing to discriminate the personage contained in it.

During the present century, the sarcophagus of King John has also been examined. It contained little else than a disorganized mass of earth. The principal substances found, were some half decayed bones, a few vestiges of cloth and leather, and a long, rusty piece of iron, apparently the remains of the sword-blade of that monarch.—*The Medical World.*

*Revival of Urn-Burial:* By the Editor of the Edinburgh Medical Journal.—A curious discussion has been raised by the Académie of Médecine of Paris, on the mode of disposing of the dead. Several of the leading Paris journals, particularly the "Presse" and the "Siécle," defend with great boldness the assertion of the Académie, that the vicinity of Père la Chaise and the cemetery of Montmartre is gradually introducing new diseases amongst the working classes; and that in summer time, the hospitals are crowded with the victims of pestilence engendered by the fowl air of the graveyards in the neighborhoods of Paris. The discussion is likely to lead to some result and to become a party question; for a new journal, to be devoted entirely to this one question, has just appeared. This journal, called "La Crémation," is edited by two of the first writers of the "Presse," and is supposed to be quite in accordance with the sentiments of the government. M. Alexandre Bonneau proposes to replace all cemeteries adjoining to all great cities by an edifice to be denominated a "sarcophagus." This edifice to occupy the highest spot of ground in the city, " where the corpses of both rich and poor should be conveyed, there to be laid out on a metallic tablet, which, sliding by an instantaneous movement into a concealed furnace, would cause the whole body to be consumed in the space of a few minutes." With true French instinct, M. Bonneau proceeds to urge not only the utility to the public, but also the interests of art in this new method of disposing of the dead, for he points out with great complacency the new element of prosperity to the artists existing in the furnishing of funeral urns, which he declares would soon open a new source of expense and luxury to the rich. "For who would not love to preserve the ashes of his ancestor? The funeral urn would soon be found to replace on our consoles and mantel-pieces, the present ornaments of bronze clock and china vases now found there." All this may seem a misplaced pleasantry to English minds; but in Paris, these things find serious men to write and fight in their defence; and we cannot help feeling rather startled on reading the sanitary report which first led to their discussion. " The vicinity of the cemeteries is a constant source of mortality. No matter from what quarter the wind blows, it must bring over Paris the putrid emanations of Père la Chaise, of Montmartre, or Montparnasse; and the very water which we drink, being impregnated with the same poisonous matter, we become the prey of new and frightful diseases of the throat and lungs, to which thousands of both sexes fall victims in the spring and autumn of every year. Thus the *angine couenneuse*, which baffles the sklll of all our most experienced medical men, and which carries off its victims in a few hours, is traced to the absorption of the vitiated air into the windpipe, and has been observed to rage with the greatest violence in those quarters situate on the outskirts of the town, and, consequently, the nearest to the cemeteries."

The latter argument has created many converts to the opinions of M. Alexander Bonneau; and the first number of " La Crémation" has excited much interest. After a long interval of desuetude, Sir Thomas Brown's " Urn-burial" may come to be consulted as a work for practical details—and the urns in our museums, instead of representing obsolete utensils, may become models for those vases which present such charms for M. Bonneau. Perhaps, however, the vase theory is a step too far in advance, and the Parisians, if they see their way to consumption by fire, may prefer burying the ashes of their friends in the earth, as was done with the remains of Shelley's burned body, rather than that the dust of humanity, however rich the enclosing caskets, should be chimney ornaments in drawing-rooms.

The utilitarian character of the English, as distinguished from the more fanciful temperament of the French, is exemplified in the mode of interment adopted in the case of the late Sir William Temple, as detailed in the " Times" some days ago. The body was interred in a bed of charcoal, whilst the gases from the coffin are conducted by a pipe to the outside of the church. The leading journal speaks in high terms of the conserving influence thus exercised on the lungs of worshippers; but we are not so sanguine as to the benefits of the system. It may do in rare instances of intra-mural burial, but if universally adopted, congregations would be saved at the expense of the general public. Cremation is a process to which the British mind will not soon be reconciled, and the only graveyard reform presently within reach is distant cemeteries and deep sepulture amongst charcoal or other deodorizing substances.—*Abs. Med. Sci. No.* 24.

Dr. Londe, member of the French Academy of Medicine, has contributed to the *Revue de Thérap Méd. Chirurg.*, (Nov. and Dec., 1856,) several papers of interest upon the cemeteries of Paris in relation to the modes of interment, exhumations, hygienic influences, etc. Emanations from putrefying animal bodies, he avers, cause, when concentrated, vertigo, *malaise*, nausea, loss of appetite, fainting, asphyxia, and death. Hence, he argues against the former practice of burying saints and others under the alters of the churches—a practice, which, in 1744, a Huguenot professor of Montpellier was bold enough to oppose as not only inconvenient but dangerous to health.

In 1776 the government of France restricted the privilege of burial in the churches to a few of the higher orders of the clergy. In 1804 this practice was wholly interdicted, not only as to the churches but in regard to the densely inhabited or central districts of cities.

Interment in towns, in churchyards, within the walls and under the altars of churches, is attributable to chris ians rather than pagans. This pernicious practice, which originated in the fourth century of our æra, was directly at variance with the more salubrious method of disposing of the dead in ancient Greece and Rome, namely, cremation or burning, together with the interment of the ashes and burnt bones without the cities. The burning of the dead among the ancient Greeks was an almost invariable practice; even their monumental cenotaphs did not contain dead bodies at all. Among the Romans all the dead were not burned. Many were interred in the ground but always beyond the limits of the cities. The early christians, who were opposed to cremation, and who at first adopted the Hebrew method of burying in the earth, sometimes in vast excavations or caves, viewing the body as resting only for a time in the grave, in anticipation of "the resurrection and the life," from the best motives deposited their dead in the vicinity of churches, and finally in the churches themselves. This honor was, however, restricted to personages illustrious for their piety or position. The genius of philanthropy guided by lights of science is now directed towards the correction of this insalubrious practice, and its total abandonment may be expected at no very remote period.

Of a Parisian cemetery, *Père la Chaise*, the author of "the American in Paris" (1839) says: "thirty years ago it had only fourteen tombs." Carter, in his letters, says "that this cemetery, made by Napoleon, was opened for the first burial, May 21, 1814, and that by the year 1825, it had received one hundred thousand dead. It contains seventy acres, being three miles from the centre of the city, upon the declivity of Mount St. Louis, having rocks, hills, vales, shade," etc.

The first named work gives a summary of the regulations for the inhumation of the dead in towns and cities: "All cemeteries are required to be located beyond or without the towns, avoiding low, wet, confined situations. The dead bodies are to be covered with at least four feet of earth, with four feet interval between each, and two feet at the head and foot, about fifty-two square feet for each corpse. The graves are disposed of in perpetuity, or in temporary sessions of six years; the former at twenty-five dollars per mètre of three feet, two mètres being required for a grave, and the latter at ten dollars; these being disposable anew at the end of the term, the first purchaser having the refusal. All the funerals are in the hands of a company, who have their office, keep a register of the dead, attend all wants, carriages, grave-diggers, weepers, etc. They have a fixed price for the rich, which enables them to bury the poor for nothing."

Dr. Londe says, that although the bodies are destroyed in two years, the grants, for greater security, in this behalf, now extend to the end of the fifth year. He says that in the cemetery Montparnasse, one hundred and twenty-two thousand have been interred within the last twenty years. In fifty years it will have received five hundred thousand dead bodies.

Interment in the *fosses* (graves) is the most usual mode in Paris. Bodies buried in vaults (*caveaux*) sometimes become desiccated or mummified, in positions favorable to the drying process. Examples of this kind of mummification have sometimes, though rarely, occurred in the vaults in New Orleans. In the Catholic cemetery, No. 2, as the sexton informed the writer in 1840, there had been a vault opened in the upper tier, where was found the body of a man which had been long entombed, and which had never undergone putrefaction. It was dry, but otherwise little changed; the eyes, though desiccated, remained; the hair and whiskers, firmly set; the color of the skin, natural.

In dilapidated tombs, when the coffins had been placed on or near the ground, I found that the bones not yet wholly decomposed, might be crushed into a coarse powder by grasping them in the hand. The bones of a Frenchman aged 49, buried in 1809, and those of a man aged 22, buried in 1815, crumbled into dust from very slight pressure; of their coffins not a vestige remained.

In the vaults of the New Orleans cemeteries, a body buried in the summer is generally decomposed in three months; if buried in the winter, six months may be often required to separate the bones, and dissipate the offensive gases. At all seasons, when the weather is warm, fœted emanations are apt to abound. Owing to heat and humidity, mahogany coffins seldom last two years; those of cypress have been found perfectly sound after thirteen years.

The Delta of the Mississippi, rivals that of the Nile in whatsoever may be inconvenient and insalubrious in connection with inhumation. If cremation should ever become customary, it will be more suitable foı New Orleans than for most cities. Whether New Orleans is, or is not the best place to live in, is an open question, but there can be little doubt that it is one of the worst places for such as desire a cheap, dry, convenient, and comfortable grave.

The greatest anniversary in New Orleans is that of the First of November—All Saints' Day—when the city pours its living masses into the Catholic cemeteries, either as mourners for the dead or spectators of the gorgeous decorations of the tombs—a social feature highly characteristic of this as compared with any other city of the Republic. At this season, which is usually dry and comparatively cool, the decomposition of the dead is considerably retarded, and the offensive emanations are no longer insupportable. Yet at all seasons, offensive gases escape and spread through the air, as the dead are interred not *in but above* the ground. The defective construction of the tombs and vaults, the perviousness, porosity, elevation and fissures, and even falling of the brick walls, in which sometimes wood is used, the bad quality or temporary duration of the mortar, the humidity of the soil, and the heat of the sun, all combine to favor the escape of fœtid gases, which are at least repulsive, even should they not be so deleterious to the health of the city, as some writers have supposed. At all seasons, and at unexpected hours, mourners will sometimes visit and continue long at these tombs, pouring out the saddest lamentations, as I have witnessed, myself unperceived, while making statistical researches into monumentel histories of the dead.

Without dwelling upon the sanitary influences of the New Orleans cemeteries, it may be remarked that their moral aspect is, perhaps, conformable to the French type, as set forth by a celebrated authoress of England, whose mortal tableau of the cemetery of *Père la Chaise* will close this panorama of the tomb :

" Many groups in deep mourning were wandering among the tombs ; so many, indeed, that when he turned aside from one, with the reverence one always feels disposed to pay to sorrow, we were sure to encounter another. This manner of lamenting in public seems so strange to us ! How would it be for a shy English mother, who sobs inwardly and hides the aching sorrow in her heart's core—how would she bear to bargain at the public gate for a pretty garland, then enter amid an idle throng, with the toy hanging on her finger, and, before the eyes of all who chose to look, suspend it over the grave of her lost child ? An English woman surely must lose her reason either before or after such an act ; if it were not the effect of madness, it would be the cause of it. Yet such is the effect of habit, or rather of the different tone of manners and of mind here, that one may daily and hourly see parents, most devoted to children during their lives, and most heartbroken when divided from them by death, perform with streaming eyes these public lamentations. It nevertheless is impossible, let the manner of it differ from our own as much as it may, to look at the freshly trimmed flowers, the garlands and all the pretty tokens of tender care which meet the eye in every part of this wide-spread mass of moral nothingness, without feeling that real love and real sorrow have been at work.

" One small enclosure attracted my attention as at once the most *bizarre* and the most touching of all. It held the little grassy tomb of a young child, planted round with choice flowers, and crucifix and other religious emblems, several common play-things, which had doubtless been the latest joy of the lost darling. His age was stated to have been three years, and he was mourned as the first and only child after twelve years of marriage. Belew this melancholy statement was inscribed—

*Passants ! priez pour sa malheureuse mere !*
(Travellers ! pray for his unhappy mother !)

Might we not say that

Thought and affection, passion, death itself,
They turn to favor and to prettyness ?"

*Paris and the Parisians*, 115.

[*N. O. Med. & Surg. Journal.*

